# Wireless Remote Monitoring of Glucose Using a Functionalized ZnO Nanowire Arrays Based Sensor

**DOI:** 10.3390/s110908485

**Published:** 2011-08-29

**Authors:** Syed M. Usman Ali, Tasuif Aijazi, Kent Axelsson, Omer Nur, Magnus Willander

**Affiliations:** Department of Science and Technology (ITN), Campus Norrköping, Linköping University, SE-60174 Norrköping, Sweden; E-Mails: etausif@gmail.com (T.A.); kenax@itn.liu.se (K.A.); omeno@itn.liu.se (O.N.); magwi@itn.liu.se (M.W.)

**Keywords:** electrochemical nanosensor, ZnO nanowires, glucose oxidase, Nafion® membrane, remote monitoring, data acquisition, Global System for Mobile Communications (GSM)

## Abstract

This paper presents a prototype wireless remote glucose monitoring system interfaced with a ZnO nanowire arrays-based glucose sensor, glucose oxidase enzyme immobilized onto ZnO nanowires in conjunction with a Nafion^®^ membrane coating, which can be effectively applied for the monitoring of glucose levels in diabetics. Global System for Mobile Communications (GSM) services like General Packet Radio Service (GPRS) and Short Message Service (SMS) have been proven to be logical and cost effective methods for gathering data from remote locations. A communication protocol that facilitates remote data collection using SMS has been utilized for monitoring a patient’s sugar levels. In this study, we demonstrate the remote monitoring of the glucose levels with existing GPRS/GSM network infra-structures using our proposed functionalized ZnO nanowire arrays sensors integrated with standard readily available mobile phones. The data can be used for centralized monitoring and other purposes. Such applications can reduce health care costs and allow caregivers to monitor and support to their patients remotely, especially those located in rural areas.

## 1. Introduction

Research in the field of ZnO nanostructures and sensors has brought about real opportunities for the development of effective glucose biosensors. People have realized that integration of small and cheap microcontrollers with these sensors and nanosensor can result in the production of extremely useful devices, which can be used as an integral part of a wireless sensor network. These devices are called sensor nodes. Nodes are able to communicate each other over different protocols. Studies in the field of communication protocols for acquiring distributed data from remote locations for monitoring of health care and industrial parameters are under consideration among researchers. Each sensor device is typically capable of sensing, sampling, processing and communicating one or more physical, physiological, or biological signals from the environment where they are deployed. Health care costs are rapidly increasing due to the fast increase in the aging population around the World; the numbers of patient suffering from diabetes and old aged related diseases are also increasing. Wireless health monitoring system using health sensors in-home and out of hospital may assist residents and caregivers by providing non-invasive and invasive continuous and periodic health monitoring with minimum interaction between doctors and patients. A number of wearable physiological monitoring systems have been developed for the elderly to monitor the health status of the individual wearer [1–6]. As with an increasing cost of healthcare and a growing population of seniors in nursing homes and hospitals worldwide, patient monitoring using communication technologies is being considered as a solution to both improving the quality of healthcare and reducing the rate of increase for healthcare services [7–11]. In general, patient monitoring involves periodic transmission of routine physiological parameters and transmission of alerting signals when parameters cross a threshold, patients cross a certain boundary, or the device battery strength drops below a certain level. There are many challenges in wireless monitoring of patients, including the coverage, reliability and quality of monitoring. Handheld monitors are characterized by lightweight, low cost, and comfortable for long-term usage and easy to handle by individuals and ambulatory health monitoring, with instantaneous feedback to a user about the current health status [12]. Therefore, physiological monitor technology has recently focused on integrating personal digital assistants and miniaturizing the sensor systems, including embedded microcontrollers, wireless networking, and microphysical sensors for portable devices [13,14]. Recently, there have been big improvements in wireless communications, sensor technology, mobile computing, and the electronics industry. These technological advances have led to the emergence of a new generation of intelligent wireless sensor devices which are very small in size, light in weight, yet smart and powerful in functionality [15,16]. Modern wireless communication technology and services have made information more accessible than ever and there are more than a billion GSM subscribers in a more than 200 countries [17]. As the GSM infrastructure has proven to be reliable and cost effective, the services provided by GSM systems are inevitably used for data acquisition and monitoring applications. With the worldwide deployment of mobile and wireless networks, these wireless infrastructures can support many current and emerging health care applications.

In this paper, we have designed a prototype circuit for remotely monitoring glucose using our newly fabricated ZnO nanowires arrays-based sensor with immobilized glucose oxidase enzyme and successfully interfaced it with the existing GSM network. This proposed system can provide a means of using emerging nanosensors/nanodevices for monitoring multiple health parameters outside the traditional hospital environment and efficiently transfer data to physicians for immediate consultation in case of urgent need.

## 2. Experimental

### 2.1. Reagents

Glucose oxidase (E.C. 1.1.3.4) from *Aspergillus niger* 360 U/mg (BBI Enzymes (UK) Ltd.), Bovine serum albumin (BAS ≥ 98%), glutaraldehyde (50% solution), Nafion (5 wt.%), d-(+)-glucose (99.5%), zinc nitrate hexahydrate and hexamethylenetetramine (HMT, (C_6_H_12_N_4_)) were purchased from Sigma–Aldrich. Phosphate buffered, 10 mM solution (PBS) was prepared from Na_2_HPO_4_ and KH_2_PO_4_ (Sigma–Aldrich) with sodium chloride in 0.135 mM, the pH was adjusted to 7.4. Glucose stock solution was kept at least 24 h after preparation for mutation. All chemicals used were of analytical reagent grade.

### 2.2. Fabrication and Electrochemical Measurement of ZnO Nanowire Arrays Based Sensor

**Figure 1.**
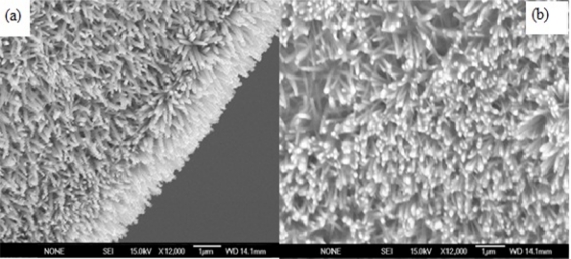
A typical scanning electron microscopy SEM image of ZnO nanowires arrays grown on 250 μm silver (Ag) wire using low temperature chemical growth. The figure shows that the diameter of the nanowires arrays is in the range of 100–200 nm. The SEM images in figure **(a)** is showing the nanowires arrays without immobilizations and **(b)** showing nanowires with enzymes immobilizations.

A 3 cm long clean, straight piece of silver wire (250 μm diameter) was first rinsed with acetone followed by rinsing in deionized water and then it was dried at room temperature. To grow ZnO nanowire arrays on the silver wire a low temperature chemical approach as described in our earlier investigations was adopted [18–20]. First the wire was dipped into a seed solution for 2 min and then dried in air. This procedure was repeated twice. The seed solution contained 0.025 M zinc nitrate and 0.025 M HMT. The solution was kept at 90 °C during the growth of the ZnO nanowire arrays. Subsequently, the wire was washed with distilled water and dried at room temperature. Typical ZnO nanowire arrays grown on the silver wires using this procedure are shown in Figure 1.

**Figure 2.**
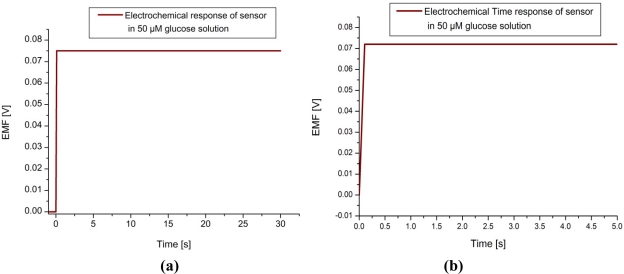

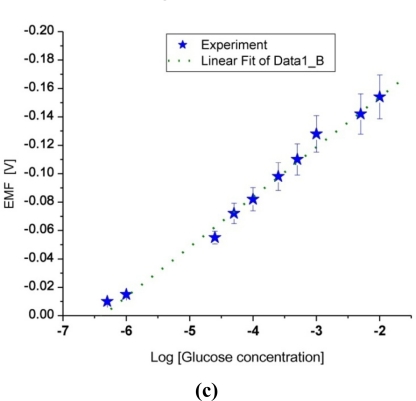
**(a)** Calibration curve of the sensor electrode showing the stable and smooth signal in 50 μM glucose solution. **(b)** Curve showing the time response of the sensor. **(c)** Calibration curve of the sensor electrode showing the electrochemical potential difference at different glucose concentrations for the ZnO nanowires arrays coated with glucose oxidase enzyme with Nafion membrane electrode with Ag/AgCl reference electrode.

As clearly seen from the SEM images, ZnO nanowire arrays of 100–200 nm diameters with uniform density and spatial distribution had been grown. These nanowire arrays were perpendicular relative to the surface of the silver wire. The morphological and structural characteristics of the grown arrays can be controlled by adjusting the growth process parameters such as the concentration of the seed solution, the reagent stoichiometry, the temperature and the pH of the growth solution [21]. To immobilize the glucose oxidase (GOD) on the ZnO nanowire array-coated silver wire, the zinc oxide electrode was rinsed with PBS to generate a hydrophilic surface. An enzyme solution was prepared by dissolving 10 mg GOD and 20 mg BSA in 500 μL PBS and the electrode was dipped into enzyme solution for 15 min and then left in air for 2 h to dry. The cross-linking procedure was carried out by adding 2 μL aqueous solution containing 2.5% glutaraldehyde and 0.5% Nafion onto the electrode surface. After drying at room temperature, an additional 2 μL of 0.5% Nafion solution was applied onto the electrode surface to prevent possible enzyme leakage and eliminate foreign interferences. All enzyme electrodes were stored in dry condition at 4 °C when not in use. After completing these steps, the sensors were initially checked potentiometrically in 100 μL of 100 μM glucose solutions with an Ag/AgCl reference electrode. The electrochemical responses of the sensors (EMF) are quite stable DC voltages with a fast response time as shown in Figure 2(a,b), and negligible noises were observed during all the experiments. The tested sensor configuration showed large dynamic ranges, with an output response (EMF) that was linear *vs.* the logarithmic concentration of glucose going from −10 mV for 0.5 μM and −154 mV for 10 mM glucose, as shown in Figure 2(c). This corresponds to a slope around 35 mV/decade and this potentiometric response of the nanosensor in terms of electrical signal was coupled using flexible shielded cables and interfaced with the designed circuits using instrumentation amplifier.

## 3. Results and Discussion

### 3.1. Study of Interferences and Temperature

The selectivity of a glucose sensor depends on two major factors that are the enzyme–analyte reaction and selective measurements. The enzyme–analyte reaction is very specific due to the nature of the enzyme glucose oxidase functionality. As a result of this reaction δ-gluconolactone and hydrogen peroxide are produced. These two products and the oxygen consumption can be used for the glucose determination. With the H_2_O availability in the reaction, gluconolactone is spontaneously converted to gluconic acid, which at neutral pH, form the charged gluconate^−^ and proton (H^+^) products according to the Equations (1) and (2) given below:

(1) $$H_{2}O+O_{2}+\beta \text{-D-glucose}\overset{\text{Glucose}\;\text{oxidase}}{\to }\;\;\;\;\delta \text{-gluconolactone}+H_{2}O_{2}$$

(2) $$\delta \text{-gluconolactone}\overset{\text{spontaneous}}{\to }\;\;\;\;{\text{gluconate}}^{-}+H^{+}$$

**Figure 3.**
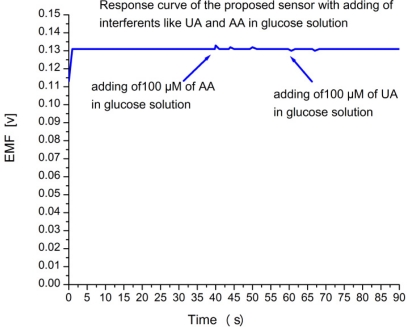
Calibration curve showing the study of interferences with time trace line of output response (EMF) change with time after adding 100 μM ascorbic acid and uric acid in 1 mM glucose solution.

**Figure 4.**
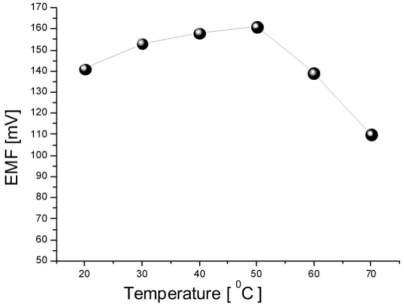
Calibration curve illustrated the influence of the temperature on the sensor response.

The glucose oxidase reaction with β-D-glucose is highly specific without any major interfering reaction with other types of sugars. It could however, be useful to check possible interferences from reducing agents such as ascorbic and uric acid, which are well known interferents with amperometric glucose measurements methods. As clearly seen from the output response of the sensor, the addition of these potential interferents does not change the signal substantially. Addition of 100 μM of ascorbic acid or uric acid to 1 mM glucose only generated some extra noise, as shown in Figure 3. We suggest that the good selectivity of the present biosensor can be attributed to the permselective (charge-exclusion) property [22,23] of the Nafion films coated on the electrode. The influence of the varying temperature on the sensor response was also examined between 20 °C and 75 °C. As shown in Figure 4, the EMF response gradually increases with the increasing temperature and reaches its maximum value at around 50 °C. This is because each enzyme has maximum activity at an optimum temperature condition. After 50 °C, the response decreases, this is caused by the natural thermal degradation of the enzyme. Although the sensor showed a maximum response at 50 °C, room temperature (23 ± 2) °C is still chosen for this work in order to prevent possible solution evaporation at higher temperatures and for ease of operation.

### 3.2. Communication Technologies for Health Caring

There are different short-range and long-range wireless communication technologies which could be used in a healthcare system for the remote monitoring of physiological parameters such as Bluetooth, Ultra Wideband (UWB), ZigBee and Wi-Fi which is a WLAN (Wireless Local Area Network) and GSM/GPRS technologies [24–32]. Due to the current advances in communication technologies, a lot of research efforts are being done in the area of health care monitoring and service providing [33]. Providing health care facilities to highly populated areas as well as remote rural areas would require enormous funding for upgrading the existing infrastructure. The health care costs in the developed countries are rapidly increasing due to the substantial increase in the elderly population. Monitoring the daily physical activities can be a key to evaluating the actual quality of life of the elderly. We believe that the overall health and wellness of the elderly population can greatly benefit from the use of current communication technologies [34]. These technologies allows the fabrication of miniaturized sensors (e.g., ZnO based nanosensors) to be integrated with microcontrollers which transform this collected data from sensor into useful information by using appropriate software to connect to the outside world using wireless links.

### 3.3. Motivation for the Choice of GSM/GPRS

Today, cellular mobile communication systems like Global System for Mobile Communications (GSM), General Packet Radio Service (GPRS), EGDE, CDMA-2000, WCDMA, 3G UMTS, just to name a few, are examples of standard technologies that are widely deployed worldwide and many devices (e.g., cellular phones, smart box, laptops, *etc*.) already utilize their services. These systems are commonly known as Wide Area Networks (WANs), since they usually cover a large geographical space and offer valuable services (including voice, text, data and multimedia) to a huge number of subscribers within their geographical coverage. They are also representing the backbone networks for many other types of smaller networks like Wireless Sensor Networks (WSN), Personal Area Networks (PAN) and Wireless Body Area Networks (WBAN). These systems can themselves provide a range of valuable services to their users, such as real-time monitoring of physiological parameters like heart rates, glucose levels, blood oxygen saturation, *etc*. and providing direct feedbacks to the users, for example, via a graphical user interface (display or LCD), or a vocal user interface (e.g., speaker). By interconnecting these systems with the other existing public networks, like cellular mobile networks and the Internet, the possibilities and applications of these systems will become virtually endless. Examples of such applications include remote monitoring of patients, elderly people, athletes, or soldiers via the Internet. As another example, we can consider a situation where a patient suffering from chronic disease (e.g., heart attack, diabetic, *etc*.) possesses healthcare monitoring systems (like ECG, glucose sensor, *etc*.). If the remote monitoring system is connected to a mobile phone network, then the system can alert the hospital (or caregiver/doctor), e.g., via SMS, once anomalies are detected (e.g., heart rate has exceeded a predefined threshold, or a certain insulin level decline). As the GSM/GPRS infrastructures have proven to be reliable and cost effective, the services provided by these systems are inevitably used for data acquisition and monitoring applications. Thus, we have chosen this system in our present work due to its wide area coverage and can reach to doctor/caregiver at any time.

### 3.4. Working Description and Block Diagram of Remote Glucose Monitoring System

**Figure 5.**
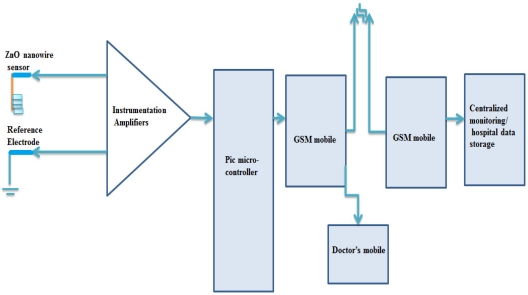
The proposed system block diagram of wireless remote monitoring system for the functionalized ZnO nanowire arrays based glucose sensor.

Figure 5 shows the block diagram of the proposed wireless remote monitoring system for the ZnO nanowires based glucose sensor. The electrical signals generated by our glucose sensor are stable, as shown in Figure 2(a), and strong enough, ranging from 10 mV to hundreds of mV with varying glucose concentrations. These signals were firstly collected by electrodes, then passed through an amplifier and filters to get rid of noises using flexible shielded cables.

After that the signals are connected to the input port of the built-in ADC of the PIC18F452 microcontroller. The PIC18F452 is a general purpose MCU from Microchip. It has several convenient built-in modules such as A/D, UART, SPI, PSP, *etc*., which reduce development time. In particular, an on-chip analog-to-digital converter (ADC) eliminates the need to design separate ADC circuitry. Also, its on-chip universal asynchronous receivers transmit (UART) module makes asynchronous communication with any compatible transceiver module relatively simple.

**Figure 6.**
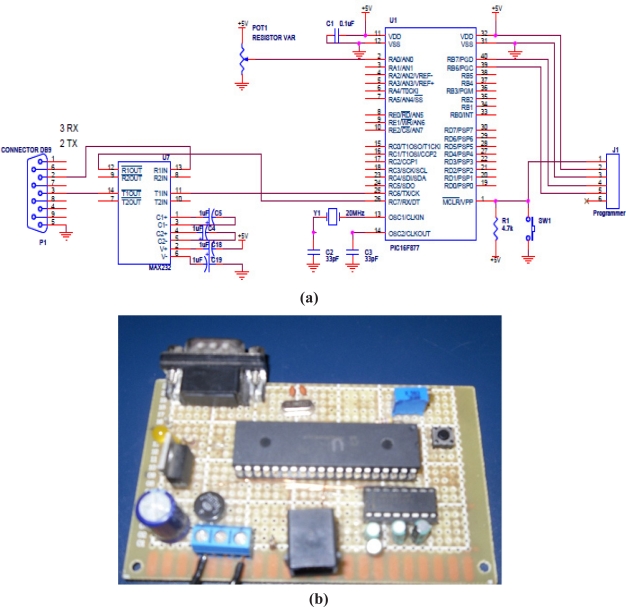
**(a)** The proposed system circuit diagram and **(b)** photograph of the designed prototype circuit board.

The ADC and UART modules operate in parallel with the microcontroller’s CPU. Operation of A/D and UART modules in parallel with the CPU saves CPU instruction cycles. In other words, once the ADC and UART are configured properly, they operate almost independently with the CPU inside PIC Microcontroller. The system schematic diagram and the photograph of the designed prototype circuit board are shown in Figure 6(a,b).

The input signal from the sensor is first interfaced to an instrumentation amplifier using flexible coaxial cables whose gain was accordingly adjusted to an appropriate level and then this signal amplified by instrumentation amplifier (IA). The output from the amplifier is then fed to the input of the ADC built in with microcontroller which converts this signal into the corresponding digital signals readable by the microcontroller. Software in the controller, which is written in C language, then reads the signal and compares it to a lookup table. This algorithm has the key responsibility of reading the sugar level in terms of input electrical signal and from the lookup table converts it into corresponding molar concentrations. After conversion it generates an instruction set for the GSM mobile device connected with RS 323 serial data cable to the circuit board. This instruction set, when passed to the mobile using the serial port, sends the sugar levels as SMS messages to the physician’s mobile and medical data storage system for immediate consultation and medication. The SMS message typically takes 10 to 30 s to deliver, but depending on the network load, it may take longer than 30 s. In this study a previously developed communication protocol [32] is used for the monitoring of sugar levels. The system detected the sugar levels in modeled glucose solutions and sent an SMS message as designed. During the test, the SMS delay was found to vary between 8 and 30 s. The system is also tested in manual simulation mode and similar results were obtained.

## 4. Conclusions

We have successfully demonstrated utilization of existing GSM/GPRS infrastructure for remotely monitoring the glucose levels with our newly fabricated ZnO nanowire arrays-based glucose sensor. This new combination of nanotechnology (nanosensors/nanodevices) with existing communication infrastructures has a promising potential to offer a wide range of benefits to patients, caregivers and the society for the monitoring of abnormal conditions and supervision of early rehabilitation. The proposed ZnO nanosensor device with immobilized glucose oxidase enzyme coated with a Nafion membrane showed good linearity and negligible interference effects on the sensor response of anionic species like uric acid and ascorbic acid. The calibration curve of the glucose concentrations is linear from 0.5 μM to 10 mM, with a detection limit of 0.5 μM. Further studies are underway to develop a user friendly miniaturized handheld multichannel nanosensor device capable of simultaneously monitoring different physiological parameters with our proposed system using the wireless communication technologies.
